# A 60-year-old man with chronic renal failure and a costal mass: a case report and review of the literature

**DOI:** 10.4076/1752-1947-3-7285

**Published:** 2009-08-04

**Authors:** Germán Campuzano-Zuluaga, William Velasco-Pérez, Juan Ignacio Marín-Zuluaga

**Affiliations:** 1 Department of Internal Medicine, Hospital Pablo Tobón Uribe, Calle 78B No. 69-240, Medellín, Colombia

## Abstract

**Introduction:**

Brown tumors are a rare focal manifestation of osteitis fibrosa cystica, which results from hyperparathyroidism. Chronic kidney failure may lead to secondary or tertiary hyperparathyroidism and thus to osteitis fibrosa cystica and brown tumors.

**Case presentation:**

A 60-year-old man with a history of diabetes mellitus and chronic kidney failure presented with a 15-day history of dyspnea, cough, malaise and fever. Initially, there was little correlation between his history and his physical examination. Various pulmonary, cardiac and infectious etiologies were ruled out. A chest X-ray showed a costal mass that was further verified by tomography and gammagraphy. The mass was suspected of being neoplastic. After a failed biopsy, the mass was removed surgically and on histopathology was compatible with a giant-cell tumor versus a brown tumor caused by hyperparathyroidism. Laboratory tests showed elevated calcium, phosphate and parathyroid hormone concentrations. The patient was diagnosed with a brown tumor secondary to refractory hyperparathyroidism.

**Conclusion:**

Tending towards a diagnosis because it is more frequent or it implies more risk for the patient may delay the consideration of other diagnostic options that, although rare, fit well into the clinical context. The patient presented here was suspected to have an osseous neoplasia that would have had major implications for the patient. However, reassessment of the case led to the diagnosis of a brown tumor. Brown tumors should be an important diagnostic consideration in patients with chronic kidney failure who have secondary or tertiary hyperparathyroidism and an osseous mass.

## Introduction

The first case in the literature reporting a brown tumor was published in 1953 and described a fronto-ethmoidal brown tumor [1]. However, previous reports of patients with localized forms of osteitis fibrosa cystica (OFC) suggest that the clinical entity was described earlier, at a time when there were few treatment options for chronic kidney failure (CKF) and consequently chronic hyperparathyroidism was more prevalent. Brown tumors are rare osseous lesions that represent a focal manifestation of OFC resulting from hyperparathyroid states. Patients suffering from CKF may develop secondary or tertiary hyperparathyroidism due to altered phosphorus and calcium metabolism. Persistent hyperparathyroidism leads to altered osseous metabolism with bone resorption and tissue changes collectively known as OFC. Our case report describes a patient with poorly controlled CKF who presented with a non-specific clinical picture and no clear diagnosis. Incidentally a costal mass was found and the diagnostic workup that followed led to an unexpected diagnosis.

## Case presentation

A 60-year-old man was transferred from the hemodialysis unit to the emergency room because of a 15-day history of malaise, subjective fever, shortness of breath, dry cough, abdominal pain and diarrhea. He also complained of mild anterior thoracic pain not associated with other symptoms and which was not irradiated. He had a 20-year history of type 2 diabetes mellitus (DM) that required insulin, with micro- and macro-vascular complications such as diabetic retinopathy and CKF. He was on hemodialysis and had a history of multiple failed dialysis accesses. He also suffered from arterial hypertension, upper and lower extremity peripheral arterial disease, carotid artery disease, a first degree atrioventricular heart block and had smoked one packet of cigarettes per day for the last 20 years. He was being treated with sevelamer, erythropoietin, folic acid, lovastatin, gemfibrozil, NPH insulin, amlodipine and acetylsalicylic acid, but was not receiving calcium or a vitamin D supplement.

A physical examination revealed the patient to be in a fair condition, with no apparent distress, hydrated, alert and well oriented. He had a heart rate of 92 beats per minute, respiratory rate of 14 breaths per minute, blood oxygen saturation of 97%, arterial blood pressure of 130/70 mmHg and no fever. He had bilateral blindness and mild epistaxis through the left nostril. The thorax was tender to palpation in some costochondral unions, but pain was poorly localized. The vesicular murmur had reduced intensity and no pathologic sounds were auscultated. Peripheral pulses were weak in both the upper and the lower limbs. He had a translumbar hemodialysis catheter. The remaining physical examination was unremarkable.

**Figure 1:**
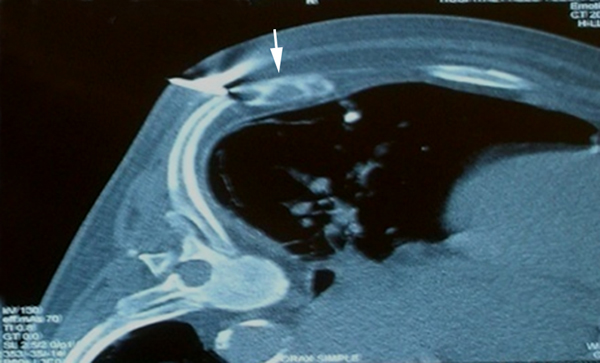
**Tomographic image during guided biopsy procedure**. Note the heterogeneous 4 × 1.3 cm mass (arrow), with preserved cortical bone and no periosteal reaction or other inflammatory signs. No cysts were identified.

The patient had stable vital signs and had no signs of systemic inflammatory response. However, because of the patient's previous history of DM, CKF and the presence of leukocytosis, neutrophilia and elevated C-reactive protein upon admission (Table 1), we initially ruled out a gastrointestinal or lung infection, or any cardiac cause for the patient's symptoms. The electrocardiogram showed no signs of ischemia, and the chest X-ray showed cardiomegaly, a small left pleural effusion, a circular opacity in the right inferior thoracic region and no signs of consolidation. These findings were initially interpreted as a pulmonary infection, probably a lung abscess, an abscedated nodule or pulmonary tuberculosis. A contrast tomography scan of the chest was ordered for further characterization. Though it showed no parenchymal compromise, a 4 × 1.3 cm lesion was observed on the right dorsal region of the eighth rib. The lesion showed thinning of cortical bone in some areas, preserved cortex and lacked periosteal reaction (Figure 1). The radiology staff considered a bone metastasis as a first diagnostic option, and a thoraco-abdomino-pelvic tomography scan was done in search for more lesions and a probable primary tumor. Additional hypodense lesions were observed, including one on the left lamina of L4, acetabulum, and head and neck of the right femur. There was no lymph-node or internal organ compromise. A Tc 99 m Medronate osseous gammagraphy reported a hypermetabolic focus compatible with a neoplastic lesion, concordant in size and location with the costal mass reported in the previous imaging studies. It also revealed generalized osseous compromise compatible with renal osteodystrophy and did not confirm the other lesions described on tomography. A tomography-guided biopsy specimen (Figure 1) was obtained, but histopathological analysis reported normal tissue components.

**Table 1:** Laboratory results upon admission and throughout hospital stay

Laboratory exams (reference range)	Day 1	Day 2	Day 7	Day 9	Day 18	Day 20
Hemoglobin (13.5-17.5 g/dl)	8.7		6.7	8.2	7	7.8
Platelets (150-350×10^3^/mm^3^)	456		559			
Leukocytes (4.5-11×10^3^/mm^3^)	14.5		10			
Neutrophils (40-70%)	82		70			
Lymphocytes (22-44%)	2		14			
CRP (0.08-3.1 mg/liter)	12.20					
Creatinine (<1.5 mg/dl)	6.5					
BUN (10-20 mg/dl)	43					
Sodium (136-145 mg/dl)	137				140	139
Potassium (3.5-5 mg/dl)	5.0				7.4	5.9
Calcium (9-10.5 mg/dl)	11.2	9.5			9.1	9.2
Phosphorus (3-4.5 mg/dl)	5.3					
Alkaline phosphatase (30-120 U/liter)		139				
Troponin* (0-0.4 ng/ml)	1.83	1.19				
Fecal occult blood		Negative				
PSA (0-4 ng/ml)		0.748				
CEA 0-3.4 (μg/liter)		3.6				

* Chronic renal failure may elevate troponin levels independently from myocardial damage.

**Figure 2:**
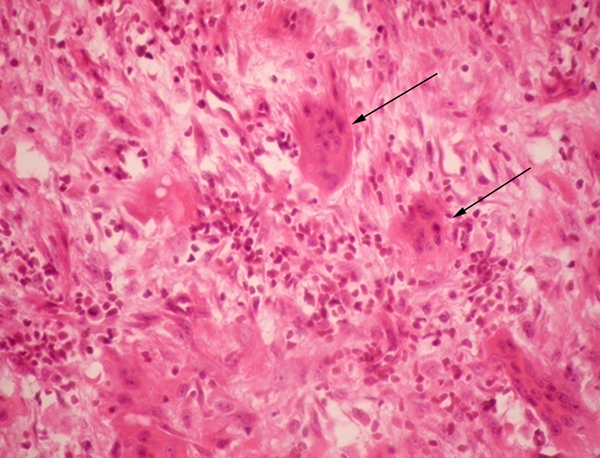
**Microscopic pathology of surgical specimen**. Presence of various multinucleated giant cells (arrows) and spindle arranged cells. Hemosiderin deposits were not observed in the sample. Hematoxylin-eosin stain at 40 × magnification.

Not being able to reach a clear diagnosis, a careful reassessment of the patient's clinical record led to considering the alternative diagnosis of renal osteodystrophy. This was supported by a history of poorly controlled CKF, elevated calcium (11.2 mg/dl) and phosphorus (5.3 mg/dl) concentrations, a phosphocalcic product of 59.36 mg^2^/dl^2^, and a bone gammagraphy that showed changes compatible with OFC. However, the possibility of neoplasia was still being considered so the mass was removed surgically. Histopathological studies reported an osseous tissue with spindles of fusiform cells in a storiform disposition with abundant multinucleated giant cells, some macrophages and some mononuclear cells. Scarce mitotic activity was observed, and there were no signs of malignancy (Figure 2). The pathologist concluded that the findings were compatible with a giant-cell tumor or a brown tumor, both histologically very similar [2]. Parathyroid hormone (PTH) concentration was 1377 pg/ml. These findings were compatible with refractory hyperparathyroidism, and a diagnosis of a brown tumor of hyperparathyroidism associated with CKF was reached.

The patient continued ambulatory medical treatment with vitamin D, calcium and sevelamer. Two months after discharge, the parathyroid level was 1900 pg/ml and a Tc 99 m Sestamibi scan revealed hyperfunctioning glands despite aggressive pharmacological treatment. Serum calcium and phosphorus levels were within normal limits, 9.4 mg/dl and 3.4 mg/dl, respectively. At the time of writing, the patient was awaiting parathyroidectomy as definite treatment for tertiary hyperparathyroidism associated with severe renal osteodystrophy.

## Discussion

Brown tumors are unusual bone lesions that represent a localized manifestation of OFC induced by hyperparathyroidism, independent of its cause. Increased PTH levels and locally produced tumour necrosis factor α and interleukin 1 (IL-1) by marrow monocytes induce the proliferation and differentiation of pluripotent bone-marrow cells into osteoblasts. These cells produce granulocyte macrophage colony stimulating factor, IL-6, IL-11 and stem-cell factor that induce the migration and differentiation of monocytes into osteoclasts, increasing the number of the latter in the bone tissue. Enhanced activity of osteoclasts and osteoblasts leads to bone resorption and a reduction of bone mineral concentration with an increased proliferation of fibrous tissue and extracellular matrix [3]. Brown tumors develop in 3% to 4% of patients with primary hyperparathyroidism and in 1.5% to 1.7% of patients with secondary causes of hyperparathyroidism [4]. However, around half of patients with CKF may develop OFC due to secondary hyperparathyroidism making brown tumors more frequent in these patients. Brown tumors have been reported in patients with primary hyperparathyroidism due to adenomas [5] and carcinomas [6] of the parathyroid gland; vitamin D deficiency due to lack of sunlight exposure [7] or due to intestinal malabsorption syndromes [8]; and secondary [9] or tertiary hyperthyroidism [10] in patients suffering CKF. Hyperphosphatemia with hypocalcemia caused by tubular damage and impaired vitamin D metabolism explains hyperparathyroidism in these patients.

Brown tumors are either mono- or polyostotic benign masses, painless and usually found incidentally. However, they may cause tissue damage to adjacent structures and compressive manifestations such as pain, neuropathies [11] and myelopathy [12]. The majority of cases report the maxilla and mandible as the main sites of occurrence [9]. Other common sites are the clavicles, scapula, pelvis and ribs; however, these lesions may appear in any osseous structure [7], including chondral tissue [13]. They are associated with an increased risk of fractures if localized in weight-bearing areas [14].

Brown tumors arise from foci of OFC and represent a reparative bone process rather than true neoplastic lesions, as there is no hyperplasia or clonal cell proliferation. Typical histopathology describes spindle cells or fibroblasts in areas of osseous lysis, multinucleated giant cells (probably osteoclasts), increased vascularization and accumulation of hemosiderin-laden macrophages, with micro-hemorrhages which confer a brownish appearance to the affected tissue. Cysts and areas of necrosis may be found [2,5]. Brown tumors are histologically similar to giant-cell tumors, giant-cell regenerative granulomas, cherubism and aneurismatic osseous cysts [2,4].

On X-ray imaging, brown tumors appear as lytic lesions with thinned cortical bone that may be fractured. Concurrent changes that suggest OFC such as osteopenia, a "salt-and-pepper" bone appearance, subperiosteal bone resorption and disappearance of the lamina dura around the roots of the teeth, may help differentiate it from other entities [4]. Tomographic imaging shows an osseous mass, with no cortical disruption, no periosteal reaction or inflammatory signs, a heterogeneous center and areas that suggest cysts [14]. Magnetic resonance imaging (MRI) shows variable intensities on T2-weighted images and intense enhancement on T1-weighted contrast MRI. MRI may be better for determining the presence of cysts or fluid filled levels; a finding that is very suggestive of a brown tumor [14]. Osseous gammagraphy is not indicated for the diagnosis of brown tumors; however, isolated hypermetabolic lesions or simultaneous hypercaptation of bone lesions and parathyroid adenomas, when done with Tc 99 m Sestamibi, have been described [15].

Although differential diagnoses for an isolated bone lesion are extensive, when confronted with a patient with CKF, an osseous mass and laboratory data that show increased levels of calcium, phosphate, phosphocalcic product as well as alkaline phosphatase, it is imperative to determine PTH levels to rule out hyperparathyroidism. Histopathological analysis of the osseous lesion is needed to confirm the diagnosis of a brown tumor. In the case presented here, parathyroid levels were not assessed earlier because another diagnosis, osseous neoplasia, was suspected which posed major prognostic value and risk for the patient. A parathyroid hormone measurement six months earlier reported 570 pg/ml; thus, it is probable that the pathological process evolved during this brief time.

Treatment of brown tumors relies on a definitive control of the underlying hyperparathyroid state. In a patient with CKF, this is achieved through the administration of phosphorus chelators, and calcium and vitamin D supplementation. In patients presenting with tertiary hyperparathyroidism, parathyroidectomy may be required. Osseous lesions usually cease to grow, then shrink and eventually ossify without further consequences for the patient. Surgery is required under certain circumstances, such as: 1) compressive neurologic symptoms over peripheral nerves, cauda equina or spinal medulla; 2) a significant anatomical deformity; 3) risk of a pathologic fracture; 4) when the symptoms or pain do not resolve despite adequate medical treatment and control of the hyperparathyroid state; and 5) when the biopsy does not yield a clear diagnosis, as with the present case [9,11,12].

## Conclusion

The case presented here illustrates how brown tumors, though rare, should be considered in patients with CKF and an osseous mass. The initial clinical presentation of this patient, a history of DM with a non-compensated CKF and the laboratory studies suggested an infectious process. Retrospectively, these initial complaints and findings could be explained by the patient's renal condition with volume overload, severe anemia, hydro-electrolyte disturbances, as well as altered calcium and phosphate metabolism. Early diagnosis and proper management of CKF enable an optimal control of bone-mineral metabolism, thus decreasing the incidence of OFC and making brown tumors rare lesions. Nevertheless, when confronted with a patient with CKF and an osseous mass, a brown tumor caused by hyperparathyroidism should always be considered in the differential diagnosis.

## Abbreviations

CKF: chronic kidney failure; DM: diabetes mellitus; IL-1: interleukin 1; IL-6: interleukin 6; IL-11: interleukin 11; MRI: magnetic resonance imaging; OFC: osteitis fibrosa cystica; PTH: parathyroid hormone.

## Consent

Written informed consent was obtained from the patient for publication of this case report and accompanying images. A copy of the written consent is available for review by the Editor-in-Chief of this journal.

## Competing interests

The authors declare that they have no competing interests regarding this case report.

## Authors' contributions

GCZ summarized and interpreted the patient's medical record and was part of the medical staff, did the literature review and wrote the manuscript. WV and JIMZ helped to interpret the patient's medical record, were part of the medical staff and helped to write and review the manuscript. JIMZ was the principal attending physician and responsible for most medical decisions and interpretations expressed in the article. All authors read and approved the final manuscript.
